# Comparative Effectiveness of Bevacizumab versus Cetuximab in Metastatic Colorectal Cancer Patients without Primary Tumor Resection

**DOI:** 10.3390/cancers14092118

**Published:** 2022-04-24

**Authors:** Yi-Chia Su, Chih-Chien Wu, Chien-Chou Su, Meng-Che Hsieh, Ching-Lan Cheng, Yea-Huei Kao Yang

**Affiliations:** 1 Department of Pharmacy, Kaohsiung Veterans General Hospital, Kaohsiung 813414, Taiwan; jia.love70@vghks.gov.tw; 2 School of Pharmacy, Institute of Clinical Pharmacy and Pharmaceutical Sciences, College of Medicine, National Cheng Kung University, Tainan 701401, Taiwan; 3 Department of Nursing, Shu-Zen Junior College of Medicine and Management, Kaohsiung 821004, Taiwan; 4 Division of Colorectal Surgery, Department of Surgery, Kaohsiung Veterans General Hospital, Kaohsiung 813414, Taiwan; pauleoswu@vghks.gov.tw; 5 Department of Surgery, School of Medicine, National Yang Ming Chiao Tung University, Taipei 112304, Taiwan; 6 Clinical Innovation Center, National Cheng Kung University Hospital, National Cheng Kung University, Tainan 701006, Taiwan; chienchou.su@gmail.com; 7 Department of Pharmacy, National Cheng Kung University Hospital, College of Medicine, National Cheng Kung University, Tainan 701006, Taiwan; 8 Department of Hematology and Oncology, E-Da Cancer Hospital, Kaohsiung 824005, Taiwan; philips115@gmail.com; 9 College of Medicine, I-Shou University, Kaohsiung 824005, Taiwan; 10 Health Outcome Research Center, National Cheng Kung University, Tainan 701401, Taiwan

**Keywords:** metastatic colorectal cancer, bevacizumab, cetuximab, primary tumor resection

## Abstract

**Simple Summary:**

The current metastatic colorectal cancer guidelines suggest intensive systemic chemotherapy with a targeted agent, rather than surgical resection, as first-line treatment for primary colorectal tumor and distant metastasis. However, results of comparative efficacy between bevacizumab and cetuximab remain controversial. This study aimed to assess the effectiveness of both therapies in patients who did not undergo primary tumor resection. Among patients treated with targeted agents, primary tumor resection was associated with lower mortality among those who received both bevacizumab and cetuximab. Among patients that did not undergo primary tumor resection, multivariable analysis for conversion surgery showed that the cetuximab group had a significantly higher metastasectomy rate. In these patients, cetuximab-based therapy was associated with significantly better survival compared to bevacizumab-based therapy. Cetuximab also yielded a higher conversion surgery rate.

**Abstract:**

Primary tumor resection may be unfeasible in metastatic colorectal cancer. We determined the effects of bevacizumab and cetuximab therapies on survival or conversion surgery in patients with metastatic colorectal cancer who did not undergo primary tumor resection. This retrospective cohort study enrolled 8466 patients who underwent first-line bevacizumab- or cetuximab-based therapy. We analyzed the data of both therapies in patients who did not undergo primary tumor resection. Overall survival after targeted therapy plus chemotherapy was assessed. The groups were matched using propensity score matching and weighting. Cetuximab resulted in lower mortality than bevacizumab (hazard ratio (HR) = 0.75); however, it did not have the same effect in patients that underwent primary tumor resection (HR = 0.95) after propensity score weighting. Among patients treated with targeted agents, primary tumor resection was associated with lower mortality among those who received both bevacizumab (HR = 0.60) and cetuximab (HR = 0.75). Among patients that did not undergo primary tumor resection, multivariable analysis for conversion surgery showed that the cetuximab group (HR = 1.82) had a significantly higher metastasectomy rate. In these patients, cetuximab-based therapy was associated with significantly better survival compared with bevacizumab-based therapy. Cetuximab also yielded a higher conversion surgery rate. These findings demonstrate the importance of stratification by primary tumor resection in the application of current treatment guidelines and initiation of future clinical trials.

## 1. Introduction

Colorectal cancer is a common malignancy worldwide and one of the most common causes of cancer-related mortality [1]. Globally, approximately 20–25% of patients with colorectal cancer present with metastasis at time of initial diagnosis, with the liver and lung being the most common sites of distant metastases [2].

Recently, the mortality rate of metastatic colorectal cancer declined owing to improvements in early detection and advances in comprehensive treatment, particularly the combination of chemotherapy and targeted monoclonal antibodies. Bevacizumab is a monoclonal antibody that binds to the vascular endothelial growth factor, while cetuximab acts against the epidermal growth factor receptor, and both are approved first-line treatments for metastatic colorectal cancer. Chemotherapy with 5-fluorouracil (5-FU)/leucovorin (LV)/oxaliplatin or 5-FU/LV/irinotecan combined with targeted monoclonal antibodies is the most frequently selected first-line treatment regimen for metastatic colorectal cancer [3,4]. Currently, the National Comprehensive Cancer Network guideline (version 4, 2020) suggests intensive systemic chemotherapy [5,6,7,8] with a targeted agent (such as bevacizumab or cetuximab), rather than surgical resection, as first-line treatment for primary colorectal tumor and distant metastasis.

However, results of the comparative efficacy between bevacizumab and cetuximab remain controversial. In the Cancer and Leukemia B and Southwest Oncology Group (CALGB) 80405 study, overall survival did not significantly differ between groups treated with cetuximab and bevacizumab [9]. The results of the trial conducted by a multidisciplinary team (FIRE-3 trial) demonstrated significantly better OS in the cetuximab group than in the bevacizumab group [10]. Notably, in the FIRE-3 and CALGB 80405 studies, 20% of patients did not undergo primary tumor resection prior to targeted therapy. In studies with real-world data, 39% of patients with metastatic colorectal cancer did not undergo primary tumor resection [11]. Furthermore, as a predictive effect of tumor sidedness, chemotherapy plus anti-epidermal growth factor receptor (EGFR) antibody compared with chemotherapy alone or chemotherapy plus bevacizumab had a great effect, with the effect being greatest in patients with left-sided tumors [12]. Therefore, further investigation of the difference in survival outcomes between cetuximab and bevacizumab would inform clinical decision-making for patients with metastatic colorectal cancer who did not undergo primary tumor resection, and the difference in survival outcome between right-sided and left-sided tumors in Asia. Furthermore, metastasectomy for initially unresectable tumors with subsequent conversion to resectable tumors following neoadjuvant therapy with targeted agents can achieve better survival [7,8,13]. To the best of our knowledge, no previous study has examined which targeted agent can successfully achieve conversion surgery.

The prospective randomized control trial Japan Clinical Oncology Group (JCOG) 1007 reported that primary tumor resection was not beneficial to asymptomatic patients with metastatic colorectal cancer, compared to those on systemic therapy alone [14]. van der Kruijssen et al., in another randomized control trial, demonstrated that patients with mCRC who were randomized to primary tumor resection followed by systemic treatment had higher 60-day mortality than patients treated with systemic therapy [15]. Both studies enrolled metastatic colorectal patients, with or without few clinical symptoms, who were then treated with bevacizumab-based systemic therapy. They revealed that upfront primary tumor resection was not beneficial to this population. Therefore, comparing the difference in survival outcome between bevacizumab and cetuximab in mCRC patients may further inform clinical decision-making in selecting targeted therapy.

In Taiwan, expenditure for bevacizumab or cetuximab combined with chemotherapy as first-line treatment for patients with metastatic colorectal cancer was reimbursed by the National Health Insurance (NHI) since 1 June 2011 and 1 December 2012, respectively [11]. Herein, we assessed the effects of bevacizumab and cetuximab on survival among patients with metastatic colorectal cancer who underwent conversion surgery or did not undergo primary tumor resection. Moreover, we analyzed the effects on survival outcomes due to primary tumor resection (with or without) and targeted therapy (bevacizumab or cetuximab) in patients with metastatic colorectal cancer.

## 2. Materials and Methods

### 2.1. Data Source

The NHI database (NHID) is a claims database derived from the NHI, a nationwide single-payer insurance program that covers > 99.99% of the entire Taiwanese population [16,17]. The database was used to collect complete records of the prescriptions of targeted agents, such as bevacizumab and cetuximab, chemotherapy type, and information on the surgical status of the patients. The NHID claims were thoroughly examined for all patients to determine the time of initiation and discontinuation of any targeted therapy and chemotherapy for managing metastatic colorectal cancer.

The Taiwan Cancer Registry (TCR) database has an excellent coverage rate (97%), and the quality of data from the cancer registry has been deemed excellent [18]. Information extracted from the TCR database included the date of diagnosis, severity, grade, morphologic type of cancer, tumor stage, nodal stage, tumor size, origin, stage at diagnosis, lymph node status, radiotherapy, surgical procedures, and tumor patterns. Cause of death data were evaluated to obtain mortality data and were traced to 31 December 2019. The data were anonymized. The study protocol was approved by the Institutional Review Board of Kaohsiung Veterans General Hospital (KSVGH21-CT2-03).

### 2.2. Study Population

We conducted a retrospective cohort study of newly diagnosed patients with metastatic colorectal cancer between 1 January 2006 and 31 December 2017 to identify patients in the TCR database who underwent treatment with bevacizumab or cetuximab as first-line targeted treatment. The index date was defined as the date the patient received the first cycle of bevacizumab or cetuximab during the study period. We enrolled patients who underwent at least six targeted therapy cycles, with an interval of <60 days between consecutive cycles (the list of drugs approved for treating metastatic colorectal cancer in Taiwan and the corresponding Anatomical Therapeutic Chemical Classification System (ATC) codes are supplied in Supplementary Materials). We excluded patients if they: (1) were aged < 20 years; (2) had synchronous left- and right-sided tumors; (3) underwent targeted therapy within 1 year before the diagnosis date; (4) received first-line treatment of fewer than six cycles or had a follow-up duration < 3 months; (5) received targeted therapy with intervals of >60 days between consecutive cycles; or (6) switched targeted therapy.

### 2.3. Study Variables and Targeted Therapy Exposure

Demographic variables included the year of diagnosis, year of targeted therapy, age, sex, histological grade, primary tumor location, stage (4A and 4B), tumor size, lymph node status, radiotherapy, surgical procedures before the index date (the corresponding surgical procedure codes are listed in Supplementary Materials), Charlson comorbidity index score [19,20], intra-abdominal infection (the corresponding International Classification of Diseases, ninth revision (ICD-9) and ICD-10 are provided in Supplementary Materials), and co-medication 1 year before the index date (the corresponding drug ATC codes are provided in Supplementary Materials). Additionally, mucinous (yes or no) and signet ring cell (code M-8490) (yes or no) histologic types were included in the analyses. The primary tumor location and sidedness [right-sided (appendix, cecum, ascending colon, hepatic flexure, or transverse colon) versus left-sided (splenic flexure, descending colon, sigmoid colon, rectosigmoid junction, or rectum)) were further analyzed in the subgroups. The primary outcome was overall survival, evaluated from the index date to the end of 2019, death, or censorship. Conversion surgery was defined as surgical treatment with a goal of resection in initially unresectable patients with metastatic colorectal cancer after response to systemic therapy. As a secondary outcome of conversion surgery, we followed up each patient to an event or until the end of 2019. Primary tumor resection was defined as surgery to remove the primary tumor.

### 2.4. Statistical Analysis

Descriptive statistics were calculated for demographic and tumor characteristics. A standardized mean difference exceeding 0.2 was used to identify differences in baseline covariates between the bevacizumab and cetuximab groups. Overall survival was calculated using the Kaplan–Meier method and compared using the log-rank test for unadjusted survival differences between the bevacizumab and cetuximab groups. Furthermore, we performed a subgroup analysis according to primary tumor sidedness to investigate survival outcome. However, the adjusted survival hazard ratio (HR) for comparing the two groups was estimated using multivariable analysis by fitting a Cox proportional hazards model. The results are expressed as HRs and their corresponding 95% confidence intervals (CIs). All analyses were performed using SAS version 9.4 (SAS Institute Inc., Cary, NC, USA). For all tested hypotheses, analyzed items with a two-tailed *p*-value < 0.05 indicated statistical significance.

### 2.5. Sensitivity Analyses

Five sensitivity analyses were performed to examine the results’ robustness. In the first analysis, patients receiving at least six cycles of first-line treatment with the same regimen could cause selection bias; thus, all patients received at least two first-line treatment cycles with intervals < 45 days, and the targeted agent for the first two cycles was the same. The result of the analysis was used to define the first-line targeted treatment (flow chart of cohort selection in metastatic colorectal cancer is provided in Supplementay Figure S3). In the second analysis, owing to differences in the information on different reimbursement dates in the NHID (bevacizumab and cetuximab were reimbursed for metastatic colorectal cancer on 1 June 2011 and 1 December 2012, respectively), the analysis compared the bevacizumab group with the cetuximab group after an index date in 2013. In the third analysis, we used the E-value method to assess the unmeasured confounding with *RAS* (Rat sarcoma viral oncogene homolog) mutational status association between targeted therapy and overall survival [21], because of the unavailability of *RAS* status in our database. In the fourth analysis, we evaluated overall survival considering the interaction between primary tumor resection and bevacizumab or cetuximab. Cox regression analysis was used to evaluate all-cause survival HRs for the interaction between primary tumor resection (with or without) and systemic treatment (bevacizumab or cetuximab). The attributable proportion was used to measure the fraction of the decreasing risk from the interaction between primary tumor resection and targeted therapy type [22]. In the final analysis, we included the aforementioned covariates, other than the lymph node, in a logistic regression model to generate a propensity score for patients with a probability of receiving treatment. We generated a Cox proportional hazards model adjusted for propensity score and baseline characteristics to compare the survival HRs between the two groups (Supplementary Figures S1 and S2; Supplementary Tables S1 and S2). We identified the comparison group of bevacizumab-based therapy using one-to-one propensity score matching and calculated the inverse probability of the cetuximab-based therapy group for weighting. We estimated overall survival after propensity score matching and the stabilized inverse probability of treatment weights to control confounding factors and ensure comparativeness between both groups. Potential confounders and covariates related to outcome, such as medications, comorbidities, and tumor patterns, were included in the PS model (the PS model is provided in Supplementary Materials). The stabilized inverse probability of treatment weights was used in order not to lose samples with the estimated average treatment effect.

## 3. Results

### 3.1. Cohort Characteristics

**Figure 1:**
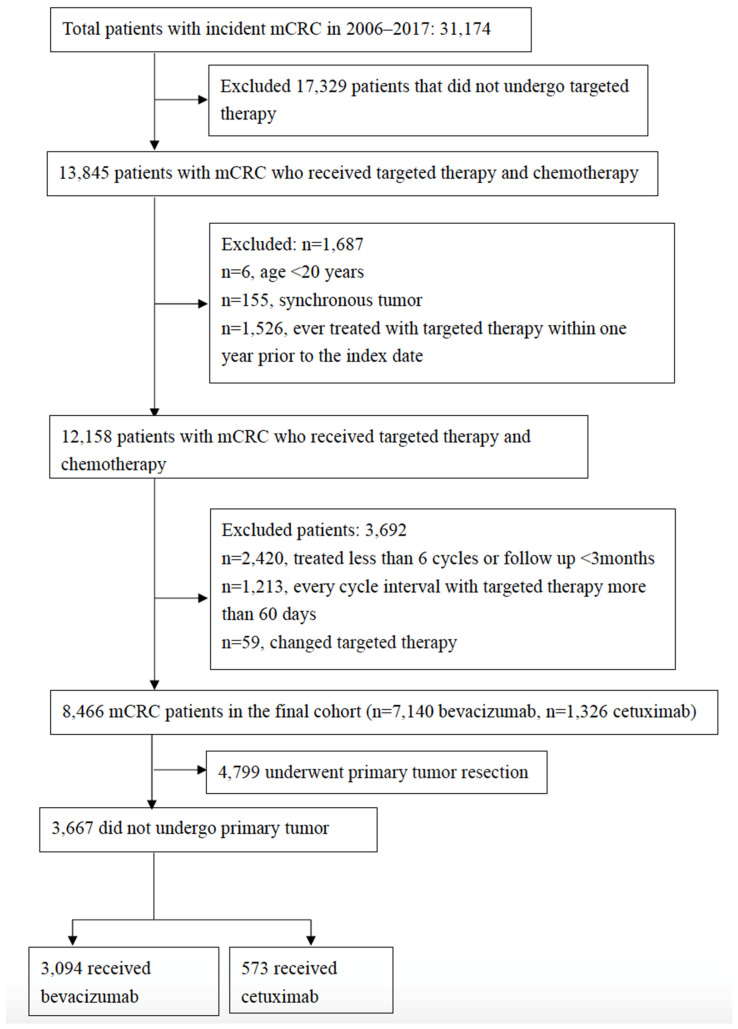
Flow chart of cohort selection. mCRC, metastatic colorectal cancer.

We identified 13,845 patients with metastatic colorectal cancer between 1 January 2006 and 31 December 2017 (Figure 1). Overall, 8466 patients who underwent targeted therapy combined with chemotherapy as first-line treatment were enrolled. There were 7140 and 1326 patients in the bevacizumab- and cetuximab-based therapy groups, respectively. Overall, 3667 (43.3%) patients did not undergo primary tumor resection prior to targeted therapy, and they comprised 3094 (43.33%) and 573 (43.21%) patients in the bevacizumab-based and cetuximab-based therapy groups, respectively. Patient characteristics are summarized in Table 1.

### 3.2. Overall Survival

**Figure 2:**
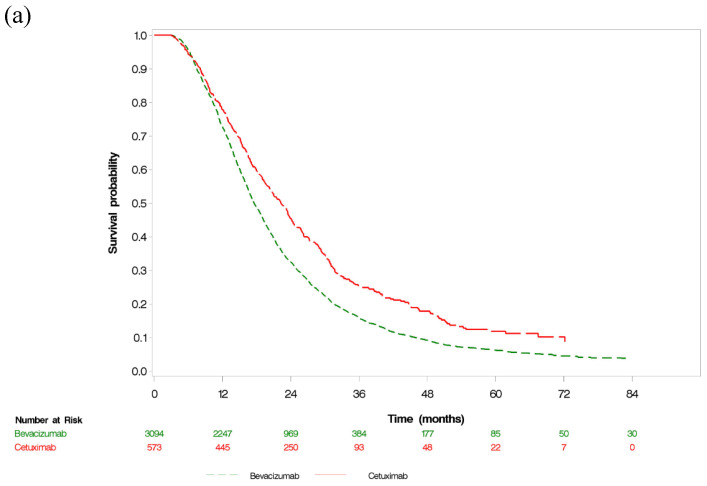

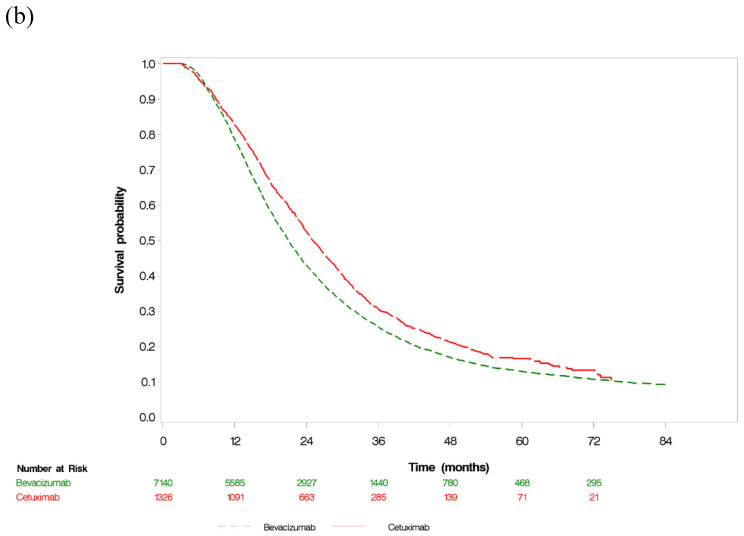
Kaplan–Meier survival curves for OS in mCRC in (**a**) patients without primary tumor resection (**b**) the overall population. mCRC, metastatic colorectal cancer; OS, overall survival.

**Figure 3:**
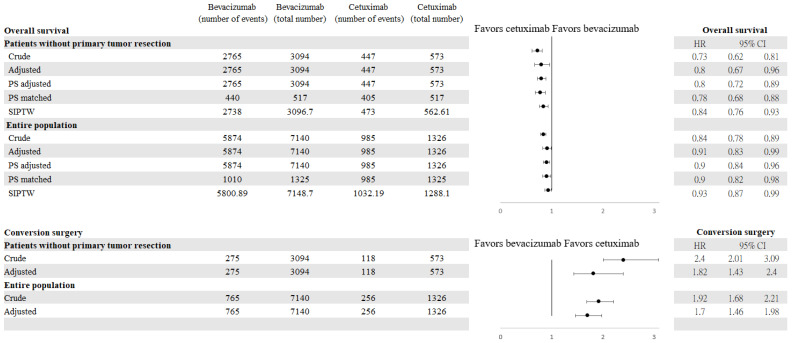
HRs of OS and conversion surgery among patients who did not undergo PTR and in the overall population; bevacizumab versus cetuximab. CI, confidence interval; HR, hazard ratio; OS, overall survival; PS, propensity score; PTR, primary tumor resection; SIPTW, stabilized inverse probability of treatment weights.

Among patients who did not undergo primary tumor resection, 3212 (87.59%) died during follow-up: 2765/3094 (89.37%) in the bevacizumab group and 447/573 (78.01%) in the cetuximab group. The median overall survival was significantly better in the cetuximab group (22.22 months; 95% CI, 20.3–23.77) compared to the bevacizumab group (17.52 months; 95% CI, 17.06–18.18), with a crude HR of 0.73 (95% CI, 0.62–0.81) and an adjusted HR of 0.80 (95% CI, 0.67–0.96) among patients who did not undergo primary tumor resection. The subgroup analysis revealed that for patients with a left-sided tumor, the median overall survival was significantly better in the cetuximab group (23.34 months; 95% CI, 21.49–25.04) compared to the bevacizumab group (18.38 months; 95% CI, 17.59–19.01), with a crude HR of 0.71 (95% CI, 0.63–0.79) and an adjusted HR of 0.75 (95% CI, 0.61–0.92) among patients who did not undergo primary tumor resection. In patients with right-sided tumors, there was no significant difference in the median overall survival between cetuximab (16.52 months; 95% CI, 11.81–20.30) and bevacizumab (15.37 months; 95% CI, 14.54–16.56) (crude HR = 0.98, (95% CI, 0.78–1.24); adjusted HR = 1.19, (95% CI, 0.80–1.78)) (Supplementary Table S5). Generally, the median OS was significantly better in the cetuximab group (24.99 months; 95% CI, 23.80–26.31) compared to the bevacizumab group (20.89 months; 95% CI, 20.53–21.32), with a crude HR of 0.84 (95% CI, 0.78–0.89). However, the HR was 0.91 (95% CI, 0.83–0.99) after adjusting for the covariates measured at baseline, leading to a significant result (Figure 2 and Figure 3). However, the rate of conversion surgery was significantly higher in the cetuximab group than in the bevacizumab group (median time to conversion surgery 15.83 and 15.44 months, respectively), with adjusted HRs of 1.82 (95% CI, 1.43–2.4) and 1.70 (95% CI, 1.46–1.98) among patients who did not undergo primary tumor resection and the overall population, respectively.

### 3.3. Sensitivity Analyses

All patients received at least two cycles of first-line targeted treatment combined with chemotherapy and cycle intervals with targeted therapy < 45 days. Among them, 4184 and 734 patients were classified as having received bevacizumab-based and cetuximab-based therapy without primary tumor resection, respectively (Supplementary Figure S3, Supplementary Table S3). The crude and adjusted HRs of overall survival associated with bevacizumab or cetuximab were 0.74 (95% CI 0.68–0.81) and 0.80 (95% CI 0.69–0.93), respectively. Conversely, in the overall population, the crude and adjusted HRs of overall survival associated with bevacizumab or cetuximab were 0.85 (95% CI, 0.80–0.90) and 0.98 (95% CI, 0.93–1.03), respectively (Supplementary Figure S3, Supplementary Table S4). After 2013, on the index date, the crude and adjusted HRs of overall survival for the bevacizumab and cetuximab groups were 0.79 (95% CI, 0.70–0.88) and 0.83 (95% CI, 0.68–1.01), respectively, among patients who did not undergo primary tumor resection (Table S3). However, the crude and adjusted HRs of overall survival for the bevacizumab and cetuximab groups were 0.89 (95% CI, 0.82–0.96) and 0.94 (95% CI, 0.86–1.04), respectively, in the overall population (Table S4). The E-value for the estimated HR and the upper confidence bound were 1.88 and 1.53, respectively, in association with an unmeasured confounder with the *RAS* profile between targeted therapy and overall survival. The observed HR of 0.78 could be explained by an unmeasured confounder that was associated with both treatment and outcome by a risk ratio of 1.88-fold each beyond the measured confounders; however, it could not be explained by a weaker confounder. The calculation was derived from the HR obtained from the matched analysis among patients who did not undergo primary tumor resection in this study. In a previous study [23], the overall survival of patients with *RAS*-wild type compared to those with *RAS*-mutant type was 1.65 (95% CI, 0.96–2.86). In the interaction between primary tumor resection and targeted therapy, the median overall survival was significantly worse in patients who did not undergo primary tumor resection and were treated with bevacizumab or cetuximab (17.52 months (95% CI, 17.06–18.18) and 22.22 months (95% CI, 20.3–23.77), respectively; *p* < 0.001) than those who underwent primary tumor resection and were treated with bevacizumab or cetuximab (24.53 months (95% CI, 23.67–25.32) and 27.37 months (95% CI, 25.65–28.89), respectively; *p* = 0.06) (Figure 2). Among all patients who received first-line systemic treatment for metastatic colorectal cancer, patients who underwent primary tumor resection had a lower mortality rate than those who did not, in both groups (bevacizumab group (unmatched: HR = 0.60, 95% CI, 0.57–0.63; propensity score matched: HR= 0.56, 95% CI, 0.50–0.64; stabilized inverse probability of treatment weights: HR = 0.60, 95% CI, 0.57–0.63); cetuximab group (unmatched: HR = 0.75, 95% CI, 0.62–0.91; propensity score matched: HR = 0.75, 95% CI, 0.55–1.01; stabilized inverse probability of treatment weights: HR = 0.75, 95% CI, 0.62–0.91)). Among patients who underwent primary tumor resection, we found no significant difference in the mortality rate between the two targeted agents (unmatched: HR = 0.91, 95% CI, 0.72–1.16; propensity score matched: HR = 1.00, 95% CI, 0.74–1.36; stabilized inverse probability of treatment weights: HR = 0.95, 95% CI, 0.75–1.20). However, patients who did not undergo primary tumor resection in the cetuximab group had a significantly higher survival rate than those in the bevacizumab group (unmatched: HR = 0.73, 95% CI, 0.66–0.81; propensity score matched: HR = 0.76, 95% CI, 0.66–0.86; stabilized inverse probability of treatment weights: HR = 0.75, 95% CI, 0.68–0.83) (Table 2). The one-third decrease in risk was attributable to the interaction between primary tumor resection and targeted therapy plus chemotherapy (unmatched: attributable proportion = 0.4; propensity score matched: attributable proportion = 0.43; stabilized inverse probability of treatment weights: attributable proportion = 0.39). Overall survival after propensity score matching and stabilized inverse probability of treatment weights between both groups yielded HRs of 0.78 (95% CI, 0.68–0.88) and 0.84 (95% CI, 0.76–0.93), respectively, in patients who did not undergo primary tumor resection (Figure 3). In the overall population, overall survival after propensity score matching and stabilized inverse probability of treatment weights between both groups yielded HRs of 0.90 (95% CI, 0.82–0.98) and 0.93 (95% CI, 0.87–0.99), respectively, in patients who did not undergo primary tumor resection (Figure 3). This finding was robust, since the results were consistent with stabilized inverse probability of treatment weights and propensity score matching.

## 4. Discussion

Our study demonstrated that patients with metastatic colorectal cancer who were unable to undergo primary tumor resection and received cetuximab treatment had significantly better survival than those treated with bevacizumab (HR = 0.73; 95% CI, 0.62–0.81), despite the relatively small sample size of cetuximab recipients. Moreover, compared with the FIRE-3 and CALGB 80405 studies [9,10], this study revealed a higher rate of patients with metastatic colorectal cancer who did not undergo primary tumor resection (43% vs. 25–15%) in the real-world setting.

Additionally, in the multivariate analysis, we found that cetuximab recipients had a higher rate of conversion surgery than bevacizumab recipients among patients with unresectable metastatic colorectal cancer (HR = 1.82; 95% CI, 1.43–2.4). This might be favorable in improving overall survival, as suggested by the post hoc analysis of the FIRE-3 trial [24]. Furthermore, an observational study [23] also found that the cetuximab group had a higher rate of conversion surgery than the bevacizumab group (29.7% vs. 25.4%), similar to that observed in this study (20.59% and 8.89% in the cetuximab and bevacizumab groups, respectively). Meanwhile, Schwartzberg et al. [25] reported better overall survival with panitumumab over bevacizumab (HR, 0.62; 95% CI, 0.44–0.89) in patients with *RAS*-WT unresectable metastatic colorectal cancer. As both panitumumab and cetuximab are anti-epidermal growth factor receptor monoclonal antibodies, our results may imply a class benefit over bevacizumab, which acts as an anti-vascular endothelial growth factor. Our study employed the NHI data sets ranging from 2006 to 2017, while panitumumab was not reimbursed until 2016. Therefore, patients treated with panitumumab would be scarce and were not included for analysis. Therefore, cetuximab may be the preferred choice as first-line treatment for patients with metastatic colorectal cancer who are unable to undergo primary tumor resection.

Patients who underwent primary tumor resection, regardless of the type of targeted therapy, experienced better overall survival than those who did not, highlighting the favorable association between primary tumor resection and survival outcomes. Our findings were similar to those of previous studies in that most patients in this study underwent surgery prior to systemic therapy [7,8,26,27,28,29,30]. However, the current prospective trials demonstrated inconsistent results. The JCOG1007 study reported that primary tumor resection did not improve survival in asymptomatic patients with mCRC but without taking account of the possible impact of tumor sidedness [14,31,32]. For the right-sided colon, the median OS was 30 months in the group with systemic therapy and only 17 months in the group with upfront PTR. On the basis of a subgroup analysis, right-sided colonic cancers had worse prognoses in the PTR followed by the chemotherapy arm [14]. van der Kruijssen et al. disclosed that upfront PTR did not affect 60-day mortality [15]. In our analyses, we performed a series of adjustments for several covariates and propensity score matching, including tumor sidedness. Moreover, the survival benefit between the cetuximab and bevacizumab groups was similar among patients who underwent primary tumor resection. These results may be owing to a dominant efficacy of primary tumor resection, which may reduce the impact of targeted therapy.

Our study demonstrated that the interaction between targeted therapy and primary tumor resection could affect overall survival. When patients were stratified into the bevacizumab- or cetuximab-based regimen groups, patients with primary tumor resection had better outcomes than those who did not undergo primary tumor resection in terms of overall survival. When stratified by primary tumor resection, overall survival in the bevacizumab and cetuximab groups was similar among patients who underwent primary tumor resection; however, the cetuximab group experienced significantly better overall survival than the bevacizumab group among patients who did not undergo primary tumor resection. Furthermore, patients in the cetuximab group who underwent primary tumor resection showed the best overall survival outcomes. This finding was robust, since the results were consistent with two propensity score matched analyses with stabilized inverse probability of treatment weights and propensity score matching. Shitara et al. showed a possible interaction between a history of previous surgery and targeted therapy in terms of overall survival (*p* = 0.097) [33].

This study showed a median overall survival of 20.89 and 24.99 months in the bevacizumab and cetuximab groups in the overall population, respectively. The HR was 0.91 (95% CI 0.83–0.99) after adjusting for covariates, which included sex, year of diagnosis, date of targeted therapy, lung and liver resection before the index date, tumor sidedness, histologic type, and staging. However, multivariate Cox regression analyses with stabilized inverse probability of treatment weights and propensity score matching performed to compare survival outcomes yielded HRs of 0.93 (95% CI, 0.89–0.99) and 0.90 (95% CI, 0.82–0.98), respectively. Particularly, this finding was consistent with that of a previous large randomized controlled trial [10] that reported a median overall survival of 25 months in the bevacizumab group and 28.7 months in the cetuximab group. The similar duration of overall survival supported the reproducibility of first-line bevacizumab or cetuximab plus chemotherapy across different countries and clinical practice settings, regardless of the chemotherapy backbone. In the subgroup analysis regarding the left-sided tumor population, our study demonstrated that patients with mCRC who received cetuximab treatment had significantly better survival than those who were treated with bevacizumab (HR = 0.75; 95% CI, 0.61–0.92); however, the patients with right-sided tumors did not exhibit this benefit. This finding was consistent with a previous randomized controlled trial [12].

The major strengths of this study include the comprehensive enrolment of patients with metastatic colorectal cancer from a nationwide claims database; the ability to capture complete information on comorbidities, treatments, procedures, and medications reimbursed by the NHI; and confirmation of diagnosis via linkage to the TCR database. Furthermore, the sample size in this study was larger than that in previous studies. These aspects helped improve the validity of our analysis and allowed us to compare the effectiveness between the two targeted biologics-based therapeutic regimens.

Our study had some limitations. First, genetic profiles were unavailable in the TCR database. Adjusted propensity score models, such as stabilized inverse probability of treatment weights and matched analysis, were performed to produce similar baseline characteristic statuses among patients receiving different targeted treatments. Therefore, this finding would be robust because the results were consistent with stabilized inverse probability of treatment weights and propensity score matching. Because of the unavailability of *RAS* profiling data, we performed sensitivity analysis for unmeasured confounding to overcome this limitation. Among patients who underwent targeted therapy, we assessed the probability in the full and sensitivity analyses by calculating the E-value to assess the association of the *KRAS* gene status between targeted therapy and overall survival. Second, because disease severity, number of metastatic organs, and the extent of metastatic disease were also unavailable for analysis, we employed the covariate of the stage (4A and 4B) to adjust for the confounding effect. Finally, there was no information on performance status, nutritional condition, length of life expectancy, hematologic information, and hepatic and renal function status in the databases. Instead, we balanced those surrogate differences between two targeted therapy groups, including age, comorbidities, and co-medication.

## 5. Conclusions

In conclusion, our findings suggest that first-line use of cetuximab combined with chemotherapy would improve overall survival, as well as the benefit of conversion surgery when compared with bevacizumab therapy in patients with metastatic cancer without primary tumor resection. This highlights the importance of stratification by primary tumor resection when applying current treatment guidelines, and for future clinical trials.

## Figures and Tables

**Table 1:** Baseline characteristics of patients with mCRC who did not undergo primary tumor resection before targeted therapy.

Characteristics	Without Primary Tumor Resection (*n* = 3667)			Overall mCRC (*n* = 8466)		
Targeted therapy	B (*n* = 3094), *n* (%)	C (*n* = 573), *n* (%)	SMD	B (*n* = 7140), *n* (%)	C (*n* = 1326), *n* (%)	SMD
Sex			0.08			0.1
Male	1805 (58.34)	356 (62.13)		4014 (56.22)	809 (61.01)	
Female	1289 (41.66)	217 (37.87)		3126 (43.78)	517 (38.99)	
Age, y			−0.08			−0.06
<50	580 (18.75)	125 (21.82)		1387 (19.43)	287 (21.64)	
50–59	865 (27.69)	162 (28.27)		1999 (28)	376 (28.36)	
60–69	862 (27.86)	154 (26.88)		2012 (28.18)	370 (27.9)	
≥70	787 (25.44)	132 (23.04)		1742 (24.4)	293 (22.1)	
Year of mCRC diagnosis			0.63			0.67
2006–2009	45 (1.45)	11 (1.92)		93 (1.3)	28 (2.1)	
2010	43 (1.39)	3 (0.52)		199 (2.79)	25 (1.89)	
2011	317 (10.25)	5 (0.87)		900 (12.61)	19 (1.43)	
2012	439 (14.19)	20 (3.49)		1042 (14.59)	60 (4.52)	
2013	432 (13.96)	89 (15.53)		997 (13.96)	211 (15.91)	
2014	423 (13.67)	125 (21.82)		917 (12.84)	261 (19.68)	
2015	405 (13.09)	92 (16.06)		880 (12.32)	225 (16.97)	
2016	467 (15.09)	95 (16.58)		1040 (14.57)	239 (18.02)	
2017	523 (16.9)	133 (23.21)		1072 (15.01)	258 (19.46)	
Year of targeted therapy			0.79			0.88
2011	290 (9.37)	0 (0)		752 (10.53)	0 (0.00)	
2012	467 (15.09)	0 (0)		1151 (16.12)	0 (0.00)	
2013	430 (13.9)	97 (16.93)		996 (13.95)	233 (17.57)	
2014	418 (13.51)	131 (22.86)		956 (13.39)	285 (21.49)	
2015	429 (13.87)	94 (16.4)		905 (12.68)	227 (17.12)	
2016	451 (14.58)	99 (17.28)		997 (13.96)	255 (19.23)	
2017	514 (16.61)	124(21.64)		1085 (15.2)	260 (19.61)	
2018–2019	95 (3.07)	28 (4.89)		298 (4.17)	66 (4.98)	
Radiotherapy	533 (17.23)	106 (18.5)	0.03	911 (12.76)	182 (13.73)	0.03
Charlson comorbidity index	2.70 ± 1.01	2.66 ± 1.05	−0.03	2.651 ± 0.98	2.62 ± 1.00	−0.03
Intra-abdominal infection	87 (2.81)	16 (2.79)	0	230 (3.22)	43 (3.24)	0
Surgery before index date						
Primary tumor resection	0 (0)	0(0)		4046 (56.67)	753 (56.79)	0
Liver resection	22 (0.71)	11 (1.92)	0.11	759 (10.63)	166 (12.52)	0.06
Lung resection	31 (1)	10 (1.75)	0.06	191 (2.68)	33 (2.49)	−0.01
Tumor sidedness			0.25			0.26
Right	810 (26.18)	89 (15.53)		2154 (30.17)	249 (18.77)	
Left	2284 (73.82)	484 (84.47)		4986 (69.83)	1077 (81.22)	
Tumor differentiation grade			0.16			0.09
Well differentiated	134 (4.33)	23 (4.01)		233 (3.26)	33 (2.49)	
Moderately differentiated	1565 (50.58)	309 (53.93)		4701 (65.84)	912 (68.78)	
Poorly differentiated	260 (8.4)	51 (8.9)		828 (11.6)	153 (11.54)	
Undifferentiated; anaplastic	16 (0.52)	3 (0.52)		85 (1.19)	15 (1.13)	
Missing	1119 (36.17)	187 (32.64)		1293 (18.11)	213 (16.06)	
Histologic type			0.19			0.2
Adenocarcinoma	2935 (94.86)	559 (97.56)		6586 (92.24)	1256 (94.72)	
Mucinous	122 (3.94)	10 (1.75)		463 (6.48)	48 (3.62)	
Signet ring cell carcinoma	37 (1.2)	4 (0.7)		91 (1.27)	22 (1.66)	
Tumor stage			0.47			0.36
T0	18 (0.58)	4 (0.7)		25 (0.35)	4 (0.3)	
T1	7 (0.23)	3 (0.52)		28 (0.39)	8 (0.6)	
T2	41 (1.33)	14 (2.44)		130 (1.82)	36 (2.71)	
T3	500 (16.16)	143 (24.96)		2538 (35.55)	533 (40.2)	
T4	342 (11.05)	66 (11.52)		2013 (28.19)	351 (26.47)	
TX	2154 (69.62)	335 (58.46)		2346 (32.86)	380 (28.66)	
Nodal stage			0.47			0.36
N0	198 (6.4)	51 (8.9)		690 (9.66)	137 (10.33)	
N1	307 (9.92)	85 (14.83)		1595 (22.34)	294 (22.17)	
N2	384 (12.41)	91 (15.88)		2420 (33.89)	498 (37.56)	
NX	2174 (70.27)	338 (58.99)		2380 (33.33)	384 (28.96)	
Tumor size			0.09			0.04
<4 cm	569 (18.39)	104 (18.15)		1633 (22.87)	313 (23.6)	
4–5 cm	373 (12.06)	74 (12.91)		1193 (16.71)	218 (16.44)	
>5 cm	966 (31.22)	197 (34.38)		2756 (38.6)	525 (39.59)	
Missing	1186 (38.33)	198 (34.55)		1558 (21.82)	270 (20.36)	
Stage			0.14			0.16
4A	1487 (48.06)	316 (55.15)		3761 (52.68)	786 (59.28)	
4B	1560 (50.42)	246 (42.93)		3284 (45.99)	512 (38.61)	
Missing	47 (1.52)	11 (1.92)		95 (1.33)	28 (2.11)	
Positive lymph nodes, number (mean ± SD)	4.80 ± 6.70	4.17 ± 5.36	−0.1	5.68 ± 6.89	5.89 ± 6.83	0.03
Chemotherapy type			0.10			0.12
No chemotherapy	3 (0.1)	4 (0.7)		16 (22)	4 (0.3)	
5-FU	121 (3.91)	11 (1.92)		281 (3.94)	26 (1.96)	
Irinotecan	8 (0.26)	3 (0.52)		16 (0.22)	11 (0.83)	
Oxaplatin	0 (0)	0 (0)		0 (0)	0 (0)	
5-FU + irinotecan	2769 (89.5)	497 (86.74)		6424 (89.97)	1169 (88.16)	
5-FU + oxaplatin	102 (3.3)	39 (6.81)		223 (3.12)	72 (5.43)	
5-FU + oxaplatin + irinotecan	91 (2.94)	19 (3.32)		180 (2.52)	44 (3.32)	
Co-medication						
Cardiac glucosides	32 (1.03)	8 (1.4)	0.03	76 (1.06)	15 (1.13)	0.01
Anti-dyslipidemia agents	531 (17.16)	107 (18.67)	0.04	1132 (15.85)	223 (16.82)	0.03
Beta blocker	681 (22.01)	153 (26.7)	0.11	1854 (25.97)	356 (26.58)	0.02
Calcium channel blockers	1031 (33.32)	187 (32.64)	−0.01	2587 (36.23)	443 (33.41)	−0.06
Diuretic agents	886 (28.64)	182 (31.76)	0.07	2489 (34.86)	465 (35.07)	0
ACEI or ARB	856 (27.67)	159 (27.75)	0	1921 (26.9)	346 (26.09)	−0.02
Anti-diabetes agents	629 (20.33)	114 (19.9)	−0.01	1577 (22.09)	304 (22.93)	0.02
Anti-hemorrhage agents	1204 (38.91)	238 (41.54)	0.05	3041 (42.59)	578 (43.59)	0.02
Anti-arrhythmic agents	405 (13.09)	91 (496)	0.08	1317 (18.45)	240 (18.1)	−0.01
Anti-fungal agents	60 (1.94)	14 (2.44)	0.03	150 (2.1)	35 (2.64)	0.04
Anti-bacterial agents	2677 (86.52)	503 (87.78)	0.04	6636 (92.94)	1225 (92.38)	−0.02
Non-selective NSAID	2119 (68.49)	366 (63.87)	−0.1	5078 (71.12)	919 (69.31)	−0.04
Selective NSAID	267 (8.63)	45 (7.85)	−0.03	578 (8.22)	109 (8.22)	0

ACEI, angiotensin converting enzyme inhibitors; ARB, angiotensin receptor blockers; B, bevacizumab; C, cetuximab; CT, chemotherapy; mCRC, metastatic colorectal cancer; NSAID, nonsteroidal anti-inflammatory drugs; SD, standard deviation; SMD, standardized mean difference.

**Table 2:** Interaction between primary tumor resection and targeted therapy in terms of overall survival for mCRC.

Operation	Bevacizumab	Cetuximab	Treatment Type within Strata of Patients with/without Operation
Un-matched			
Primary tumor resection	0.60 (0.57–0.63)	0.55 (0.41–0.73)	0.91 (0.72–1.16)
Without primary tumor resection	1 (reference)	0.73 (0.66–0.81)	0.73 (0.66–0.81)
Primary tumor resection within strata of treatment type	0.60 (0.57–0.63)	0.75 (0.62–0.91)	
PS matched ^a^			
Primary tumor resection	0.56 (0.50–0.64)	0.56 (0.37–0.87)	1.00 (0.74–1.36)
Without primary tumor resection	1 (reference)	0.76 (0.66–0.86)	0.76 (0.66–0.86)
Primary tumor resection within strata of treatment type	0.56 (0.50–0.64)	0.75 (0.55–1.01)	
SIPTW ^a^			
Primary tumor resection	0.60 (0.57–0.63)	0.57 (0.42–0.75)	0.95 (0.75–1.20)
Without primary tumor resection	1 (reference)	0.75 (0.68–0.83)	0.75 (0.68–0.83)
Primary tumor resection within strata of treatment type	0.60 (0.57–0.63)	0.75 (0.62–0.91)	

^a^ Adjusted for year of diagnosis, year of targeted index, age, sex, tumor histological grade, stage, tumor size, radiotherapies, surgical procedures before index date, Charlson comorbidity index score, intra-abdominal infection, co-medication using propensity score. Abbreviations: mCRC, metastatic colorectal cancer; PS, propensity score; SIPTW, stabilized inverse probability of treatment weights.

## Data Availability

All data generated or analyzed during this study are included in this published article and its Supplementary Information files.

## Supplementary Materials

The following supporting information can be downloaded at: https://www.mdpi.com/article/10.3390/cancers14092118/s1, Figure S1: Distribution of PS among patients without primary tumor resection; Figure S2: Distribution of PS in the overall population; Figure S3: Flow chart of cohort selection. Abbreviations: mCRC, metastatic colorectal cancer; Table S1: Baseline characteristics of the enrolled patients with mCRC without primary tumor resection treated with targeted therapy after PS matched (1:1) and PS weighted; Table S2: Baseline characteristics of the enrolled overall mCRC patients treated with targeted therapy after PS matched (1:1) and PS weighted; Table S3: HRs of overall survival among patients without primary tumor resection after PS matching (1:1) and PS weighting by targeted therapy; Table S4: HRs of overall survival in the overall population after PS matching (1:1) and PS weighting by targeted therapy; Table S5: Subgroup analysis of treatment effectiveness by tumor sidedness.
