# Supplementary material for: Comparative Effectiveness of Bevacizumab versus Cetuximab in Metastatic Colorectal Cancer Patients without Primary Tumor Resection
Source: Cancers (Basel). 2022 Apr 24;14(9):2118. doi: 10.3390/cancers14092118 (PMC9104998; doi:10.3390/cancers14092118)
Supplement: Supplementary file 1 [file cancers-14-02118-s001.zip › cancers-1695322-supplementary.pdf]

SUPPLEMENTARY MATERIALS

1. **List of Anatomical Therapeutic Chemicals (ATC) codes of drugs approved in Taiwan for the treatment of mCRC:**

Fluorouracil: ATC L01BC02
Capecitabine: ATC L01BC06
Tegafur/Gimeracil/Oteracil Pot: ATC L01BC53
Oxaliplatin: ATC L01XA03
Irinotecan: ATC L01XX19
Bevacizumab: ATC L01XC07
Cetuximab: ATC L01XC06

2. **List of surgical procedure codes in the National Health Insurance Research Database (NHIRD) for the treatment of mCRC:**

Primary tumor resection: '73011B','73012B', '73013B', '73014B', '73015B', '73017B', '73045B', '73046B','73047B','73048B', '74205B', '74206B', '74222B', '74223B', '74213B', '74214B', '74216B', '74217B', 'N26022','N26023','N26027'
Metastatic liver resection: '75002B','75003B', '75004B', '75005B', '75015B', '75016B','75017B','75018B','N26019','N26018'
Metastatic lung resection: '67023B','67042B', '67050B', '67051B', '67053B', 'N26010'

3. **Comorbidity and medication used one year before index date**

3.1 Comorbid status within one year before index date was collected in the NHIRD.
List of ICD9 and ICD10 codes of disease:
Diabetes mellitus (ICD-9 codes: 250, ICD-10: E100, E101, E106, E108-E111, E116, E118-E121, E126, E128-E131, E136, E138-E141, E146, E148, E149)
Diabetes with end organ damage (ICD-9 codes: 2504-2506, ICD-10: E107, E117, E127, E137, E147, E102-E105, E112-E115, E122-E125, E132-E135, E142-E145)
Peripheral vascular disease (ICD-9 codes: 441, 4439, 7854, V434, ICD-10: I70, I71, I731, I738, I739, I771, I790, I792, K551, K558, K559, Z958, Z959)
Heart failure (ICD-9 codes: 428, ICD-10 codes: I099, I110, I130, I132, I255, I420, I43, I50, P290)
Cerebrovascular disease (ICD-9 codes: 430-438, ICD-10: I60-I69, G45, G46, H34)
Dementia (ICD-9 codes: 290, ICD-10 codes: F00, F03, G30, F051, G311)
Chronic pulmonary disease (ICD-9 codes: 490-496, 500-505, 5064, ICD-10 codes: J40-J47, J60-J67, I278, I279, J684, J701, J703)
Connective tissue disease (ICD-9 codes: 7100,7101,7104,7140-7142,725,71481)

Myocardial infarction (ICD-9 codes: 410,412, ICD-10 codes: I21, I22, I252)
Ulcerative disease (ICD-9 codes: 5310,5317,5320,5327,5330,5337,5340,5347, ICD-10 codes: K25, K28)
Mild liver disease (ICD-9 codes:5712, 5714, 5715, 5716, ICD-10 codes: K700-K703, K709, K713, K714, K715, K717, K760, K762, K763, K764, K768, K769, Z944, B18, K73, K74, 5716)
Hemiplegia (ICD-9 codes: 342, 3441, ICD-10 codes: G81, G82, G041, G114, G801, G802, G830-G834, G839)
Moderate or severe renal disease (ICD-9 codes: 582, 585, 586, 588; ICD-10 codes: N18, N19, I120, I131, N250, Z940, Z992, 5830-5837, N032-N037, N052-N057, Z490-Z492)
Any malignant neoplasm (ICD-9 codes: 140-172, 174-195, 200-208, ICD-10: C00-C26, C60-C76, C30-C34, C37-C41, C43, C45, C58, C60, C81-C85, C88, C90-C97)
Moderate or severe liver disease (ICD-9 codes: 5722-5728, 4560-4562; ICD-10 codes: I850, I859, I864, I982, K704, K711, K721, K729, K765, K766, K767)
Metastatic solid tumor (ICD-9 codes: 196-199; ICD-10 codes: C77-C80)
AIDS (ICD-9 codes: 042-044; ICD-10 codes: B20, B21, B22, B24)
Intra-abdominal infection (ICD-9 code: 567)

3.2 We also defined information about medications used to treat these specific comorbidities from the NHIRD using the World Health Organization (WHO) ATC classification system. Patients were considered as taking these medications if there is at least 1 prescription one year before the index date.

List ATC codes of co-medications:

The medications including: beta blockers (ATC: C07), calcium channel blockers (ATC: C08, diuretics (ATC: C03), angiotensin converting enzyme inhibitors and angiotensin receptor blockers (ATC: C09), anti-diabetes mellitus agent (ATC: A10), antiplatelets (ATC: B01AC), anti-hemorrhage agent (ATC: B02), antidyslipidemic agent (ATC: C10A), antifungal agent (ATC: J02A), antibacterial agent (ATC: J01), non-Selective nonsteroidal anti-inflammatory drugs (ATC: M01AB, M01AE, M01AC), Selective nonsteroidal anti-inflammatory drugs (ATC: M01AH), cardiac glucosides (ATC: C01A), antiarrhythmics agents (ATC: C01B)

4. PS model description

We incorporated 29 variables that covered essential personal characteristics in the PS model, such as age, sex, surgery (include primary tumor resection, liver resection and

lung resection), comorbidity score (Charlson Comorbidity Index, based on the medical records one year before the initiation of targeted therapy combined with chemotherapy), concurrent medication (including calcium channel blockers, beta-blockers, diuretic agents, renin-angiotensin system inhibitors, anti-diabetes mellitus agents, dyslipidemia agents, cardiac glucoside, antiplatelet agents, anti-hemorrhagic agents, antifungal agents, antibacterial agents, antiarrhythmic agents, steroidal anti-inflammatory drugs and antibiotics) within one year before targeted therapy combined with chemotherapy initiation, tumor characteristics (include tumor laterality, tumor grading, histologic type, tumor size, stage and lymph node), year of diagnosis and index year in the PS model. We evaluated the performance of our PS model and the matching and weighting algorithm by examining the after-matching and after-weighting balance between the groups across a comprehensive list of relevant baseline covariates. The detailed distributions of baseline covariates after PS matching and after PS weighting are compared between both groups in Supplementary Table S1 (without primary tumor resection) and Supplementary Table S2 (overall) and the histogram that showed the distribution of PS within groups in Supplementary Figure S1 (without primary tumor resection) and Supplementary Figure S2 (overall).

Supplementary Table S1: Baseline characteristics of the enrolled patients with mCRC without primary tumor resection treated with targeted therapy after PS matched (1:1) and PS weighted. Abbreviations: ACEI, angiotensin converting enzyme inhibitors; ARB, angiotensin receptor blockers; NSAID, nonsteroidal anti-inflammatory drugs; SD, standard deviation

Group	(%)	Bevacizumab (n=571)	Cetuximab (n=571)	SMD	Bevacizumab (n=3096.7)	cetuximab (n=562.611)	SMD
		PS matched	PS matched		PS weighted	PS weighted	
Age	Mean	59.41	59.59	0.01	60,43	60.51	0.01
	SD	12.66	12.43		12.46	12.33	
Gender	Female	34.5	38	-0.07	41.12	41.4	-0.01
	Male	65.5	62		58.88	58.6	
Year of mCRC diagnosis	2006-2009	2.11	2.11	0.5	1.54	1.76	0.66
	2010				1.33	0.43	
	2011	5.78	0.88		9.43	0.76	
	2012	8.06	3.5		13.21	5.1	
	2013	9.81	15.59		13.25	21.82	
	2014	12.26	21.89		13.41	26.29	
	2015	13.31	16.11		13.27	15.39	
	2016	22.07	16.64		15.84	13.86	
	2017	26.62	23.29		18.72	14.59	
Index year of targeted therapy	2011	4.38	0	0.63	8.54	0	0.88
	2012	7.36	0		14.02	0	
	2013	10.33	16.99		13.15	24.33	
	2014	10.51	22.94		13.14	28.06	
	2015	14.89	16.46		14.01	16.32	
	2016	19.79	17.34		15.22	14.73	
	2017	27.85	21.54		18.25	14.08	
	2018-2019	4.91	4.73		3.66	2.47	
Stage	4A	55.17	55.34	0.00	49.11	46.59	0.10
	4B	43.43	43.08		49.27	51.65	
	Missing	1.4	1.58		1.62	1.76	
Grade (%)	Well differentiated	3.15	3.85	0.11	4.24	4.04	0.15

	Moderately differentiated	50.09	53.94		51.11	51.18	
	Poorly differentiated and undifferentiated; anaplastic	10.86	9.46		9.02	8.44	
	Missing	31.17	32.75		35.63	36.34	
Tumor size	<4 cm	16.81	18.04	0.03	18.31	17.09	0.03
	4–5 cm	12.96	12.78		12.15	12.24	
	>5 cm	36.25	34.5		31.82	33.18	
	Missing	33.98	34.68		37.72	37.49	
Histologic feature	Adenocarcinoma	98.05	97.55	0.00	95.28	94.91	0.00
	Mucinous	0.53	0.7		1.11	0.81	
	Signet ring	1.23	1.75		3.61	4.28	
Radiotherapy	no	80.39	81.44	-0.03	82.58	82.95	-0.01
	yes	19.61	18.56		17.42	17.05	
Laterality	Left	84.06	84.41	0.14	75.45	72.71	0.05
	Right	15.94	15.59		24.55	27.29	
IAI	no	98.07	97.2	0.06	97.2	97.36	-0.01
	yes	1.93	2.8		2.8	2.64	
Lung resection before index date	no	97.72	98.6	-0.07	98.88	98.9	0
	yes	2.28	1.4		1.12	1.1	
Liver resection before index date	no	98.95	98.25	0.06	99.07	99.12	0
	yes	1.05	1.75		0.93	0.88	
Positive lymph node number (number+SD)	Mean	3.4269	4.1814	0.15	4.7277	4.288	-0.07
	SD	4.6411	5.3793		6.6562	5.294	
Charlson comorbidity index	Mean	2.6725	2.669	0.00	2.6912	2.6902	0

	SD	0.9641	1.05		1.007	1.083	
Beta blockers	no	74.26	73.56	0.02	77.29	78.37	-0.03
	yes	25.74	26.44		22.71	21.63	
Calcium channel blockers	no	69.35	67.25	0.05	66.78	68.16	-0.03
	yes	30.65	32.75		33.22	31.84	
Diuretics	no	69.35	68.13	0.03	70.86	69.42	0.03
	yes	30.65	31.87		29.14	30.58	
ACEI or ARB	no	72.33	72.15	0.00	72.28	70.96	0.03
	yes	27.67	27.85		27.72	29.04	
Anti-diabetes agents	no	79.51	80.21	-0.02	79.73	78.3	0.04
	yes	20.49	19.79		20.27	21.7	
Anti-hemorrhage agents	no	63.92	58.49	0.11	60.62	58.91	0.03
	yes	36.08	41.51		39.38	41.09	
Cardiac glucosides	no	98.95	98.6	0.03	98.92	99.16	-0.02
	yes	1.05	1.4		1.08	0.84	
Anti-arrhythmic agents	no	82.49	84.06	-0.04	86.41	85.51	0.03
	yes	17.51	15.94		13.59	14.49	
Anti-dyslipidemia agents	no	82.14	81.44	0.02	82.61	80.54	0.05
	yes	17.86	18.56		17.39	19.46	
Anti-fungal agents	no	97.9	97.55	0.02	97.99	98.21	-0.02
	yes	2.1	2.45		2.01	1.79	
Anti-bacterial agents	no	12.78	12.26	0.02	13.24	12.24	0.03
	yes	87.22	87.74		86.76	87.76	
Non-selective NSAID	no	34.5	36.08	-0.03	32.29	32.54	-0.01

	yes	65.5	63.92		67.71	67.46	
Selective NSAID	no	92.82	92.12	0.03	91.47	90.63	0.03
	yes	7.18	7.88		8.53	9.37	

Supplementary Table S2: Baseline characteristics of the enrolled overall mCRC patients treated with targeted therapy after PS matched (1:1) and PS weighted. Abbreviations: ACEI, angiotensin converting enzyme inhibitors; ARB, angiotensin receptor blockers; NSAID, nonsteroidal anti-inflammatory drugs; SD, standard deviation

Group	(%)	Bevacizumab (n=1325)	Cetuximab (n=1325)	SMD	Bevacizumab (n=7148.7)	cetuximab (n=1288.1)	SMD
		PS matched	PS matched		PS weighted	PS weighted	
Age	Mean	59.57	59.53	0.00	60,09	60.12	0.00
	SD	12.07	12.45		12.33	12.12	
Gender	Female	38.34	39.02	-0.01	43.09	43.32	0.00
	Male	61.66	60.98		56.91	56.68	
Year of mCRC diagnosis	2006-2008	1.06	0.75	0.43	0.7	0.45	0.61
	2009	0.75	1.28		0.75	0.95	
	2010	1.51	1.89		2.62	1.91	
	2011	5.58	1.43		11.62	1.5	
	2012	7.92	4.53		13.61	6.34	
	2013	10.94	15.92		13.32	22.2	
	2014	11.09	19.7		12.65	24.12	
	2015	14.72	16.98		12.59	15.97	
	2016	23.17	18.04		15.44	14.18	
	2017	23.25	19.47		16.69	12.39	
Index year of targeted therapy	2011	3.25	0	0.68	9.59	0	
	2012	8.53	0		14.97	0	
	2013	11.32	17.58		13.21	25.14	
	2014	11.25	21.51		13.03	26.48	
	2015	13.28	17.13		12.81	17.19	
	2016	22.11	19.25		14.65	15.76	
	2017	22.26	19.62		16.67	12.94	
	2018	7.62	4.45		4.61	2.3	
	2019	0.38	0.45		0.46	0.19	
Stage	4A	58.77	59.32	0.02	53.66	52.16	0.09
	4B	38.42	38.64		44.86	46.44	

	Missing	1.81	2.04		1.49	1.40	
Grade (%)	Well differentiated	2.79	2.49	0.11	3.14	3.21	0.14
	Moderately differentiated	68.3	68.75		66.31	66.63	
	Poorly differentiated	12.23	11.55		11.6	11.53	
	Undifferentiated; anaplastic	1.36	1.13		1.18	1.00	
	Missing	15.32	16.08		17.77	17.63	
Tumor size	<4 cm	24.15	23.62	0.06	22.96	22.65	0.03
	4–5 cm	17.51	16.38		16.64	16.75	
	>5 cm	38.57	39.62		38.83	39.61	
	Missing	19.77	20.38		21.57	20.99	
Histologic feature	Adenocarcinoma	95.4	94.72	0.00	92.62	91.82	0.04
	Mucinous	1.51	1.66		1.34	1.29	
	Signet ring	3.09	3.62		6.05	6.89	
Radiotherapy	no	85.81	86.26	-0.01	87.08	87.02	0.00
	yes	14.19	13.74		12.92	12.98	
Laterality	Left	81.96	81.21	0.03	71.60	70.07	0.04
	Right	18.04	18.79		27.84	29.52	
IAI	no	97.36	96.75	0.04	96.78	97.06	-0.02
	yes	2.64	3.25		3.22	2.94	
Primary tumor resection before index date	no	42.04	43.17	-0.02	43.26	43.81	-0.01
	yes	57.96	56.83		56.74	56.19	
Lung resection before index date	no	96	97.58	-0.09	97.36	97.15	0.04
	yes	4	2.42		2.64	2.85	
Liver resection before index date	no	87.85	87.55	0.01	89.02	88.59	0.01
	yes	12.15	12.45		10.98	11.41	

Positive lymph node number (number+SD)	Mean	5.63	5.90	0.04	5.64	5.98	0.05
	SD	6.80	6.84		6.89	6.66	
Charlson Comorbidity Index	Mean	2.66	2.62	-0.03	2.65	2.65	0.00
	SD	0.99	1.00		0.99	0.99	
Beta blockers	no	72.83	73.21	-0.01	73.93	74.77	-0.02
	yes	27.17	26.79		26.07	25.23	
Calcium channel blockers	no	65.28	66.57	-0.03	64.21	64.46	-0.01
	yes	34.72	33.43		35.79	35.54	
Diuretics	no	65.06	64.91	0.00	65.13	65.28	0.00
	yes	34.94	35.09		34.87	34.72	
ACEI or ARB	no	72.75	73.89	-0.03	73.19	72.24	0.02
	yes	27.25	26.11		26.81	27.76	
Anti-diabetic agents	no	76.68	77.06	-0.01	77.76	76.57	0.03
	yes	23.32	22.94		22.24	23.43	
Anti-hemorrhagic agents	no	57.13	56.45	0.01	57.23	57.06	0.00
	yes	42.87	43.55		42.77	42.94	
Cardiac glucosides	no	98.94	98.87	0.01	98.93	99.11	-0.02
	yes	1.06	1.13		1.07	0.89	
Anti-arrhythmic agents	no	80.6	81.89	0.01	81.59	81.1	0.01
	yes	19.4	18.11		18.41	18.90	
Anti-dyslipidemic agents	no	83.32	83.25	0.00	83.99	82.79	0.03
	yes	16.68	16.75		16.01	17.21	

Anti-fungal agents	no	97.96	97.36	0.04	97.83	98.08	-0.02
	yes	2.04	2.64		2.17	1.92	
Anti-bacterial agents	no	7.55	7.62	0.00	7.12	6.59	0.02
	yes	92.45	92.38		92.88	93.41	
Non-selective NSAIDs	no	31.62	30.72	0.02	29.19	30.04	-0.02
	yes	68.38	69.28		70.81	69.96	
Selective NSAIDs	no	92.38	91.77	0.02	91.79	91.82	0.00
	yes	7.62	8.23		8.21	8.18	

Supplementary Figure S1: Distribution of PS among patients without primary tumor resection

(a) Distribution of propensity scores before matching

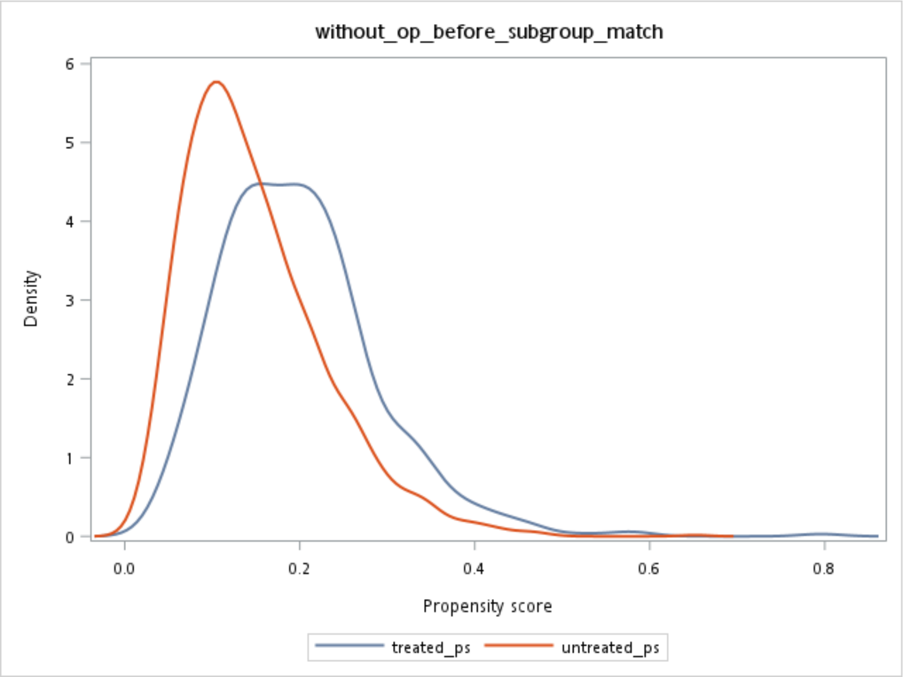

Treated_ps: cetuximab+chemotherapy; untreated_ps: bevacizumab+chemotherapy

(b) Distribution of propensity scores after matching

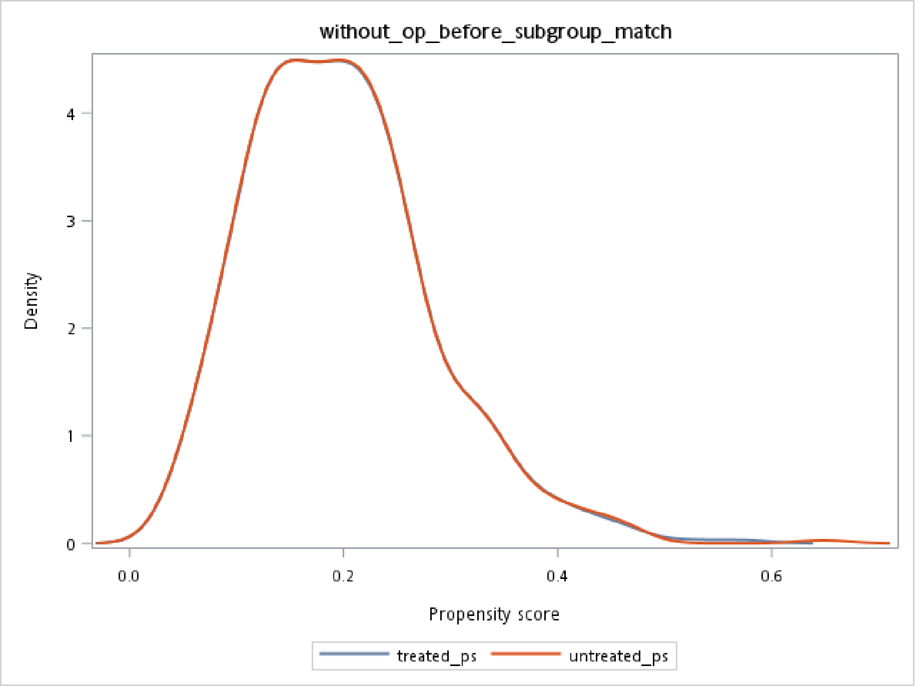

Supplementary Figure S2: Distribution of PS in the overall population

(a) Distribution of propensity scores before matching

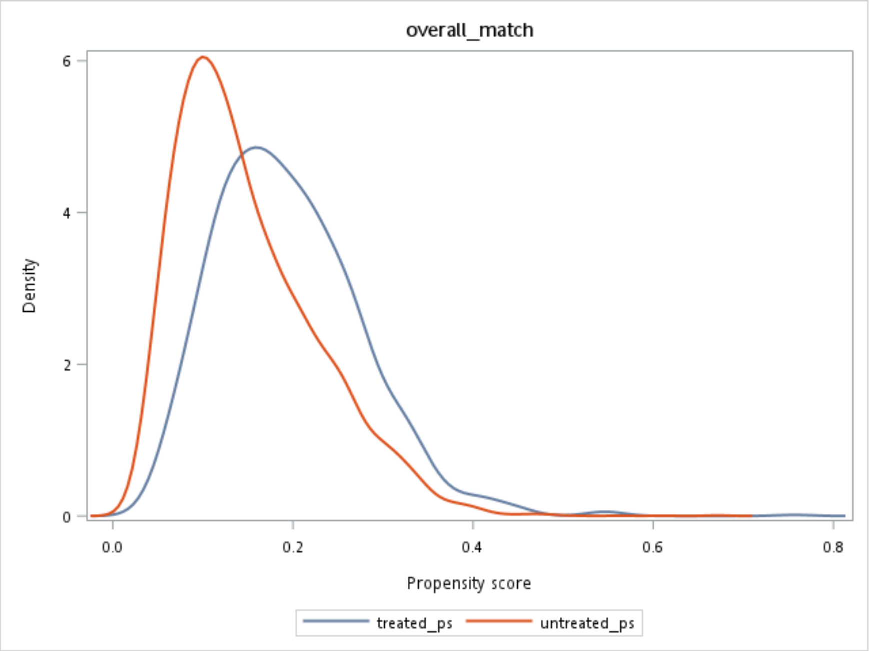

Treated_ps: cetuximab+chemotherapy; untreated_ps: bevacizumab+chemotherapy

(b) Distribution of propensity scores after matching

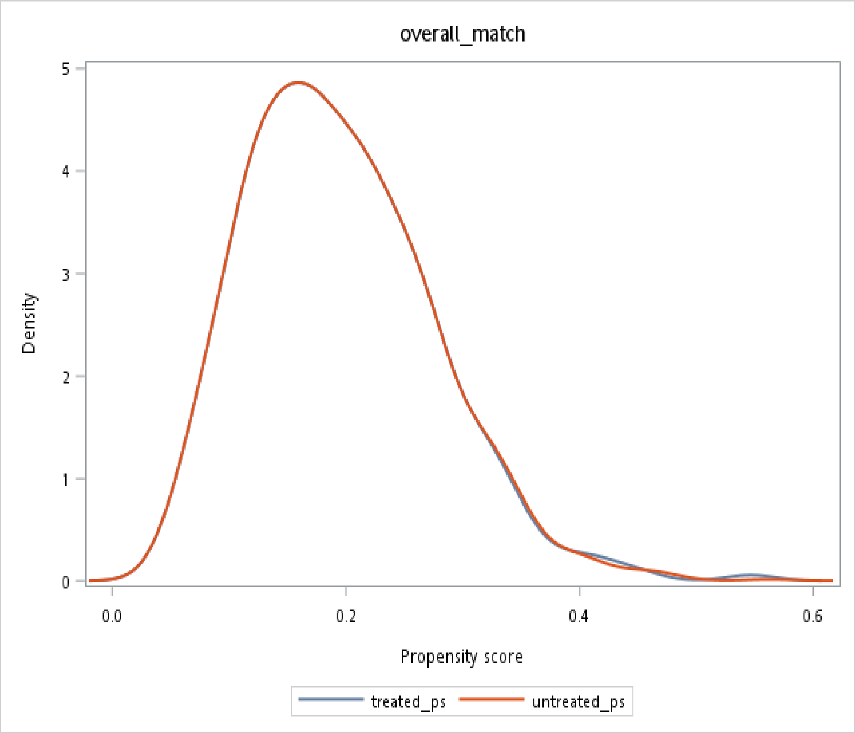

Treated_ps: cetuximab+chemotherapy; untreated_ps: bevacizumab+chemotherapy

5. Sensitivity analyses

Supplementary Figure S3 Flow chart of cohort selection. Abbreviations: mCRC, metastatic colorectal cancer

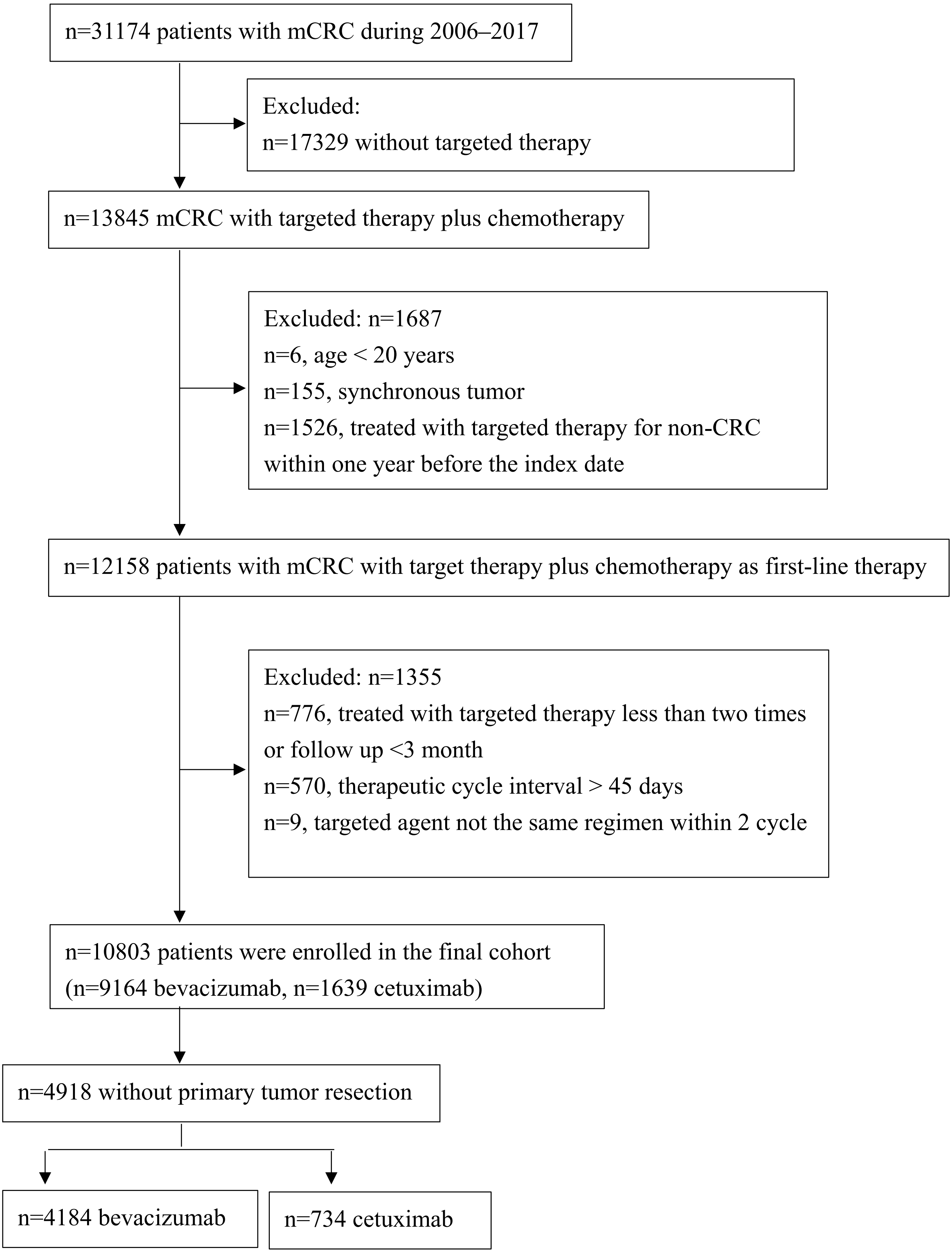

Supplementary Table S3. HRs of overall survival among patients without primary tumor resection after PS matching (1:1) and PS weighting by targeted therapy. Abbreviations: HR, hazard ratio; CI, confidence interval

	HR, 95% CI
Unadjusted (first-line targeted therapy at least 2 cycles)	0.74 (0.68–0.81)
Adjusted (first-line targeted therapy at least 2 cycles)	0.80 (0.69–0.93)
PS adjusted (first-line targeted therapy at least 2 cycles)	0.80 (0.73–0.88)
Matched and adjusted year of mCRC diagnosis and year of target therapy index (first-line targeted therapy at least 2 cycles)	0.81 (0.73–0.91)
Weighted and adjusted year of mCRC diagnosis and year of target therapy index (first-line targeted therapy at least 2 cycles)	0.83 (0.73–0.94)
Unadjusted (after index date 2013)	0.79 (0.70–0.88)
Adjusted (after index date 2013)	0.83 (0.68–1.01)
PS adjusted (after index date 2013)	0.83 (0.74–0.93)
Matched and adjusted year of mCRC diagnosis and year of target therapy index (after index date 2013)	0.76 (0.66–0.87)
Weighted and adjusted year of mCRC diagnosis and year of target therapy index (after index date 2013)	0.82 (0.74–0.91)

Supplementary Table S4. HRs of overall survival in the overall population after PS matching (1:1) and PS weighting by targeted therapy. Abbreviations: HR, hazard ratio; CI, confidence interval

	HR, 95% CI
Unadjusted (first-line targeted therapy at least 2 cycles)	0.85 (0.80–0.90)
Adjusted (first-line targeted therapy at least 2 cycles)	0.98 (0.93–1.03)
PS adjusted (first-line targeted therapy at least 2 cycles)	0.90 (0.85–0.96)
Matched and adjusted year of mCRC diagnosis and year of target therapy index (first-line targeted therapy at least 2 cycles)	0.86 (0.80–0.93)
Weighted and adjusted year of mCRC diagnosis and year of target therapy index (first-line targeted therapy at least 2 cycles)	0.93 (0.88–0.99)
Unadjusted (after index date 2013)	0.89 (0.82–0.96)
Adjusted (after index date 2013)	0.94 (0.86–1.04)
PS adjusted (after index date 2013)	0.93 (0.86–1.00)
Matched and adjusted year of mCRC diagnosis and year of target therapy index (after index date 2013)	0.88 (0.80–0.96)
Weighted and adjusted year of mCRC diagnosis and year of target therapy index (after index date 2013)	0.90 (0.84–0.97)

Supplementary Table S5 Effectiveness in tumor site subgroups by treatment

Effect	Right		Left	
	Bevacizumab	Cetuximab	Bevacizumab	Cetuximab
**Patients without primary tumor resection**				
Median OS, 95% CI, months	15.37 (14.54-16.56)	16.52 (11.81-20.30)	18.38 (17.59-19.01)	23.34 (21.49-25.04)
Number events/total number	697/774	80/87	2036/2284	365/484
Crude HR, 95% CI	0.98 (0.78-1.24)		0.71 (0.63-0.79)	
Adjusted HR, 95% CI	1.19 (0.80-1.78)		0.75 (0.61-0.92)	
**Entire population**				
Median OS, 95% CI, months	18.78 (18.05-19.64)	20.46 (17.85-22.61)	21.98 (21.29-22.55)	26.48 (25.06-27.83)
Number events/total number	1780/2110	208/246	4054/4986	774/1077
Crude HR, 95% CI	0.97 (0.84-1.12)		0.83 (0.77-0.90)	
Adjusted HR, 95% CI	0.97 (0.82-1.15)		0.89 (0.82-0.99)	
